# The Application of Closed Reduction Internal Fixation and Iliac Bone Block Grafting in the Treatment of Acute Displaced Femoral Neck Fractures

**DOI:** 10.1371/journal.pone.0075479

**Published:** 2013-09-10

**Authors:** Zhiyong Li, Wei Chen, Yanling Su, Qi Zhang, Zhiyong Hou, Jinshe Pan, Yingze Zhang

**Affiliations:** Department of Orthopaedic Surgery, Third Hospital of Hebei Medical University, Shijiazhuang, Hebei, P. R. China; Hospital for Sick Children, Canada

## Abstract

**Objective:**

This study aimed to evaluate the preliminary clinical and radiographic outcomes of acute displaced femoral neck fracture treated by closed reduction and internal fixation (CRIF) with free iliac bone block grafting with comparison to a routine protocol of CRIF without bone grafting.

**Methods:**

From December 2008 to February 2010, 220 adult patients with acute displaced femoral neck fractures were enrolled in this study. In study group, there were 124 patients (57 males, 67 females) with a mean age of 44.8 years (range, 20-64 years). There were 70 transcervical fractures and 54 subcapital fractures. The patients were treated by CRIF and free iliac bone block grafting. The control group consisted of 96 adult patients (46 males, 50 females) with a mean age of 46.3 years (range, 23-64 years). There were 61 transcervical fractures and 35 subcapital fractures. The patients in control group were treated by CRIF without bone grafting.

**Results:**

In study group, 112 patients were followed up for an average of 27.4 months (range, 24-34 months). All fractures healed within 5 months. However, 10 patients presented AVN of the femoral heads. The mean Harris score was 88.6 (range, 41-100). In control group, 68 patients were followed up for an average of 31.2 months (range, 24-42 months). The rates of AVN of the femoral head and fracture nonunion in control group were 26.5% (18/68) and 16.2% (11/68), respectively, significantly higher than those in study group (both *P*<0.05). The mean Harris score in control group was 83.8 (41–100), significantly lower than that in study group (*P*<0.05).

**Conclusion:**

Acute displaced femoral neck fractures can be treated by CRIF and free iliac bone block grafting in a minimally invasive manner. This technique can guarantee uneventful fracture healing and significantly reduce the rate of femoral head osteonecrosis.

## Introduction

Femoral neck fractures are frequently complicated by avascular necrosis (AVN) of the femoral head and fracture non-union [1]. The risk of AVN has been reported to be as high as 10% to 43% [2–10]. Non-union of femoral neck fractures has a reported incidence of 2%-22% and generally becomes apparent within 1 year [2,4,7,11–14]. The risk for non-union is greater with displaced fractures and has been reported to be as high as 30% in some series [5,8,15]. A variety of bone grafting procedures have been described to address the problem of nonunion and AVN of the femoral head, including nonvascularized bone grafting, muscle–pedicle bone grafting, vascularized bone grafting, and osteotomy [1,16–19]. However, no one technique has proved entirely satisfactory [20]. To pursue an outcome with less invasion and better functional rehabilitation, we established a protocol of closed reduction and internal fixation (CRIF) with three cannulated screws for acute femoral neck fractures, which was supplemented with non-vascularized iliac bone block graft. This study aimed to evaluate the preliminary clinical and radiographic findings of displaced femoral neck fractures treated following this protocol.

## Patients and Methods

### Ethics Statement

The study was reviewed and approved by the review board of the Third Hospital of Hebei Medical University. Signed informed consent was obtained from each patient. All clinical investigations have been conducted according to the principles expressed in the Declaration of Helsinki.

### Patients

From December 2008 to February 2010, 672 patients with femoral neck fractures were managed in our department. Adult patients with displaced subcapital or transcervical fractures were enrolled in this study. The exclusion criteria were as follows: (1) younger than 18 years or older than 65 years ; (2) with osteoporosis or pathological fractures; (3) base-of-neck fractures, which have a lower incidence of nonunion and osteonecrosis of the femoral head after CRIF [19]. Patients were divided into a study group and a control group.

In study group, patients were treated by CRIF and free iliac bone block grafting in a minimally invasive fashion. There were 124 adult patients (57 males, 67 females; 71 left, 53 right), with a mean age of 44.8 years (range, 20-64 years). The injury mechanisms included a fall from standing in 98 cases, a fall from a height in 15, a traffic accident in 10, and a crush injury in 1. There were 70 transcervical and 54 subcapital fractures. According to Garden’s classification for femoral neck fractures, the fracture pattern included 79 Type III and 45 Type IV fractures. Comminution of the posterior cortex of the neck was detected in 23 patients with radiographic evidence.

In control group, patients were treated by CRIF without iliac bone block grafting. This group consisted of 96 adult patients (46 males, 50 females; 57 left, 39 right) with a mean age of 46.3 years (range, 23-64 years). 75 fractures were caused by a fall from standing, 12 were due to a fall from a height, and 9 were caused by traffic accidents. There were 61 transcervical and 35 subcapital fractures. The fracture pattern included 64 Garden Type III and 32 Type IV fractures. Comminution of the posterior cortex of the neck was radiographically detected in 12 patients.

### Surgical Technique

**Figure 1:**
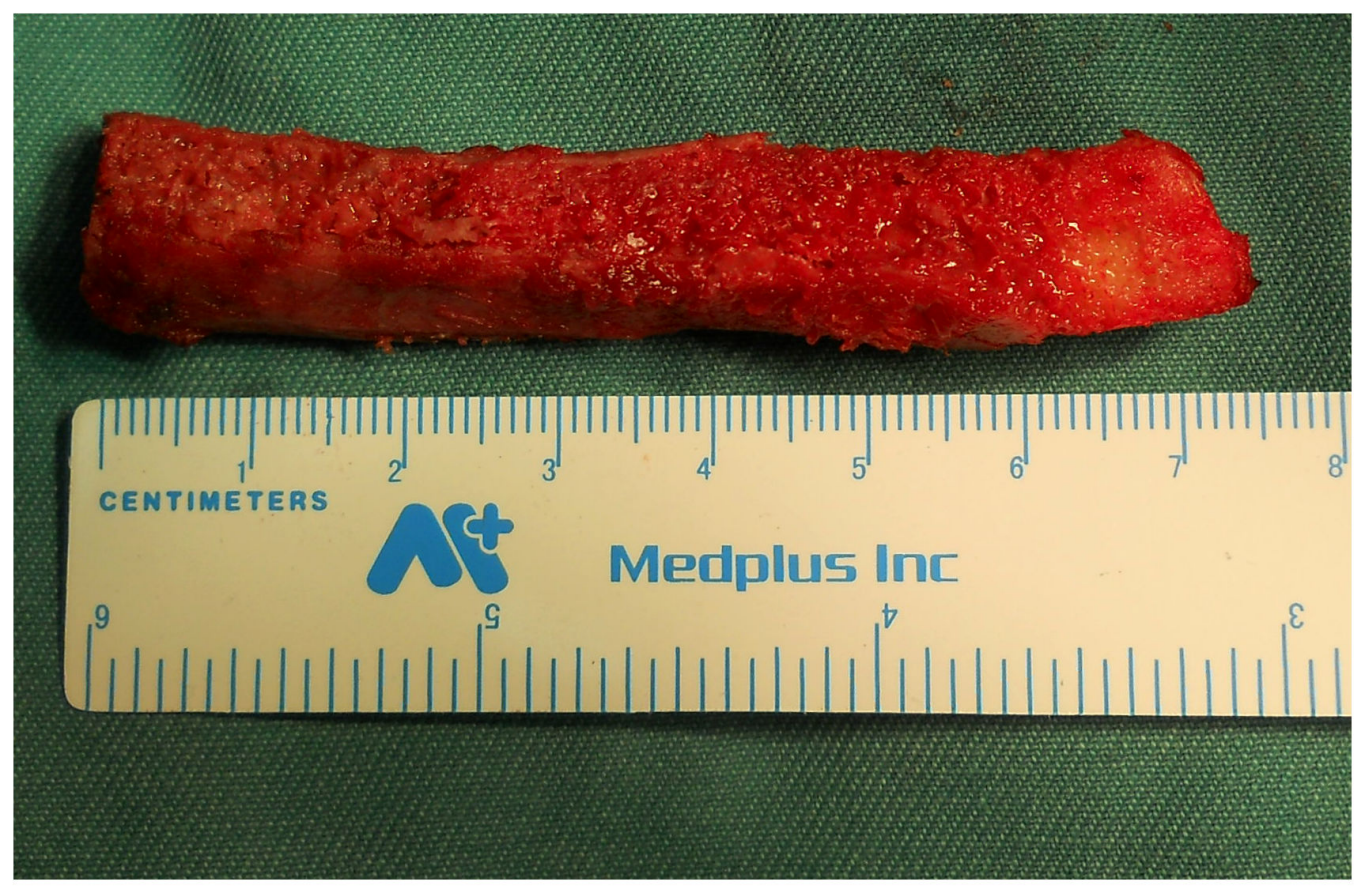
The harvested iliac bone block graft with three sides of cortex.

**Figure 2:**
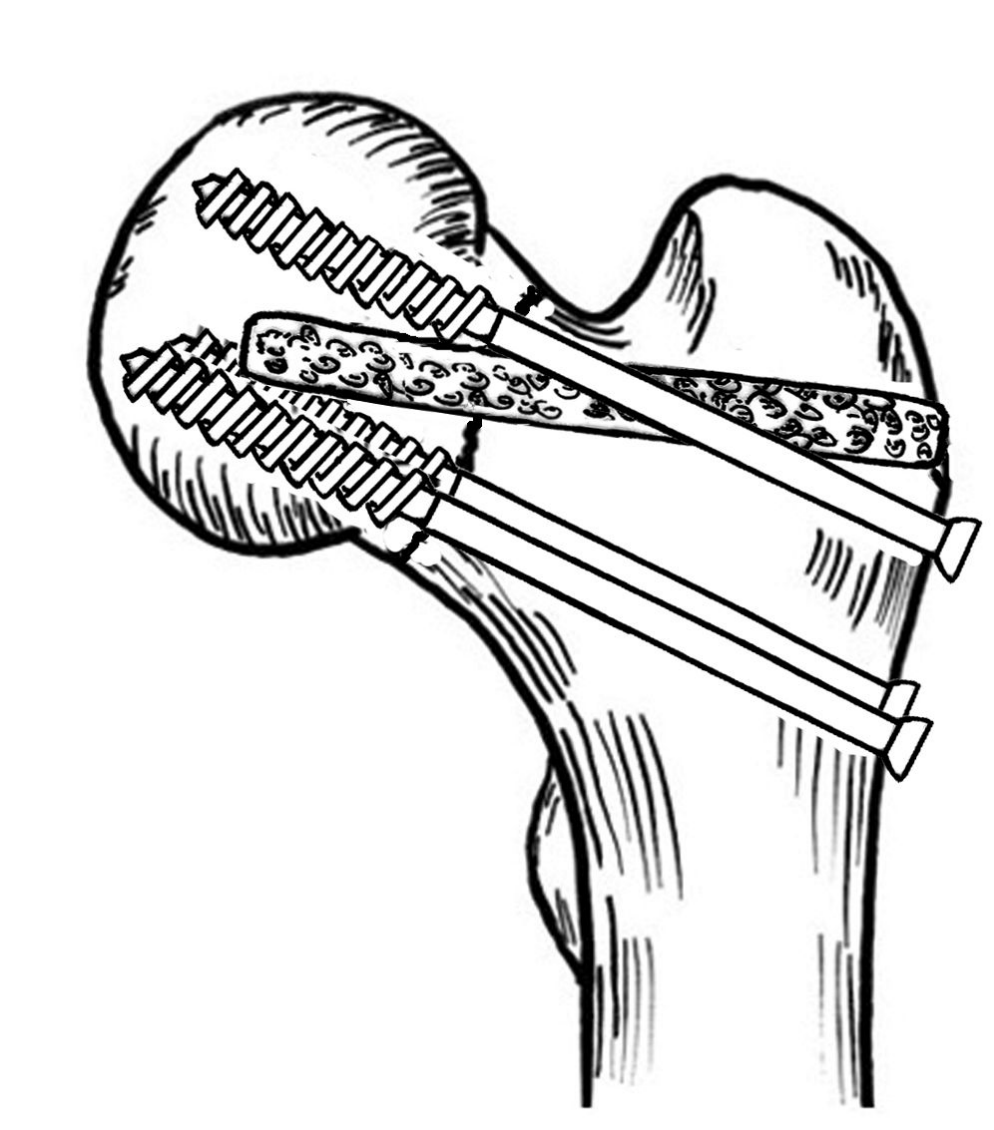
A drawing of the relative position of the iliac bone block graft and the three cannulated screws in subgroup I. The bone graft is at an angle of 30 degrees to the screws.

**Figure 3:**
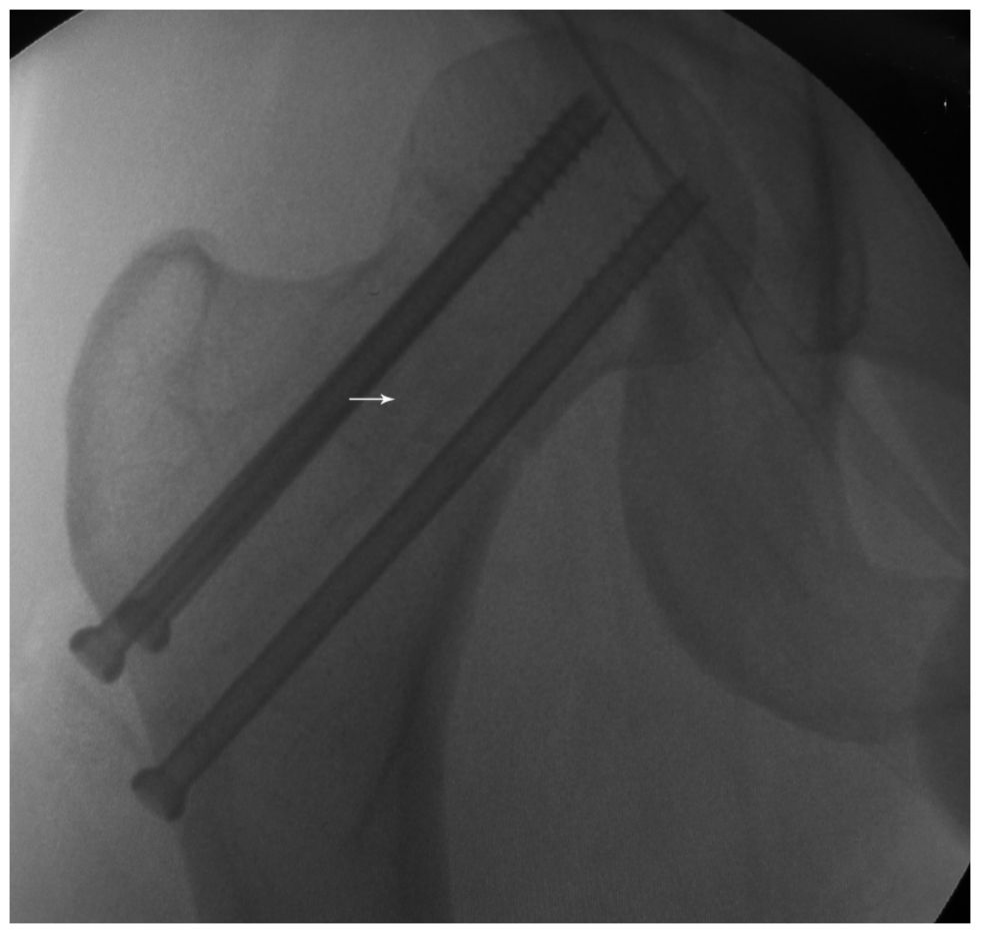
A fluoroscopic anteroposterior view of the displaced femoral neck fracture fixed with three parallel cannulated screws in subgroup II. The iliac bone block graft (arrow) is parallel to the screws.

The surgical techniques of the study group were briefly described as follows. Under general anesthesia or epidural anesthesia, patients were placed on a fracture table in a supine position. A 5-6-cm-long skin incision was made over the iliac crest. The upper surface of the iliac crest was exposed, and the abdominal muscles attached to the iliac crest were dissected from the crest. The outer surface of the iliac crest was stripped of periosteum, and a 6-8-cm-long bone block was outlined with ink. Along the marking line, a bone block 8 mm*10 mm*60-80 mm in size, including the upper, inner, and outer cortexes of the iliac crest, was harvested (Figure 1). Typically, a displaced femoral neck fracture can be treated by CRIF. Several techniques of manipulative reduction, such as Leadbetter manoeuver and Deyerler manoeuver, can be applied to reduce these fractures. If satisfactory reduction cannot be achieved by a routine closed reduction technique, the fracture can be reduced by inserting a Kirschner wire or Steinman pin into the femoral head, which acted as a joystick to manually control the movement of the proximal fragment and ensure anatomic reduction with the distal fragment [21]. The fracture was reduced anatomically or near-anatomically and fixed with three Kirschner wires, which can serve as guide wires to insert cancellous screws. In subgroup I, a fourth guide wire, at an angle of 30 degrees to the axis of the femoral neck, was inserted from the lateral cortex of the greater trochanter toward the inferior and anterior parts of the femoral head under fluoroscopic visualization. In subgroup II, a fourth guide wire, parallel to the long axis of the femoral neck, was inserted along the center of the three guide wires toward the femoral head. Then, a core tunnel 8-10 mm in diameter was created with cannulated reamers of gradually increasing size along the fourth guide pin. The size of the core tunnel was dependent on the size of the iliac bone block, which was just large enough to accommodate the graft. The graft was impacted across the fracture line until it was located beneath the subchondral bone of the femoral head. Definitive fixation was performed with three 6.5- or 7.3-mm cannulated cancellous screws along the axis of the femoral neck, which were placed triangularly with one superior and two inferior screws, that were parallel to each other. In subgroup I, the screws were placed at an angle of approximately 30 degrees to the iliac bone graft (Figure 2), and in subgroup II, the screws were placed parallel to the graft (Figure 3). All operations of the study group were performed by the senior authors (Y.Z. and J.P.) under fluoroscopic control.

In control group, the surgical technique is similar to that in study group, except that iliac bone block harvesting and grafting is not required. All operations were performed by three orthopedic surgeons with more than 15 years experience in surgical treatment of displaced femoral neck fractures.

The duration from the initial injury to operation, operative time, fluoroscopy time and blood loss were recorded and analyzed.

### Postoperative Management

Anteroposterior and lateral radiographs of the injured femoral neck were taken, and the fracture reduction was evaluated using the method proposed by Garden [22]. The patients began isometric quadriceps exercise and flexion/extension of the hip and knee joints on the first day postoperatively. The patients were encouraged to perform non-weight bearing activities for the first four weeks. This was followed by a progressive increase to full weight bearing over the following three months. All patients continued to be followed with half-yearly functional evaluations using Harris hip scores [23] as well as plain radiographic examination. Union was assessed following the criteria that the fracture gap disappeared and the patient could walk with full weight bearing without pain [19]. The Harris score and complications were recorded.

### Statistical analysis

The data were analyzed with the use of SPSS 13.0 for Windows (SPSS Inc., Chicago, IL, USA). Categorical variables were recorded as the number and percentile with frequency tables, which were analyzed using the chi-square test. The one sample Kolmogorov-Smirnov test was applied to analyze the continuous variables. Continuous variables with normal distributions were expressed as the mean±standard deviation (SD), and analyzed using the two sample *t* test. Continuous variables with non-normal distributions were recorded as the median and interquartile values, and analyzed using the Mann–Whitney *U* test. A *P*<0.05 was considered statistically significant.

## Results

### The Study Group

There were 39 patients in subgroup I and 85 patients in subgroup II (Table 1). Patient age and sex, fracture side, injury mechanism, fracture site (subcapital or transcervical) and fracture classification were compared, and no significant differences were found between subgroups (*P*>0.05). The median duration from the initial injury to operation was 2 days in subgroup I and 3 days in subgroup II. The average operative time, fluoroscopic time, and blood loss were 81 minutes, 18 seconds, and 194 mL in subgroup I, and 82 minutes, 19 seconds and 203 mL in subgroup II, respectively (all *P*>0.05). All fractures were reduced in a closed fashion and fixed with three parallel cannulated screws. Femoral neck fracture reductions were assessed on postoperative radiographs. In subgroup I, 22 reductions were assessed as Garden index grade I and 17 as grade 2. In subgroup II, 51 reductions were assessed as Garden index grade I and 34 as grade 2. There was no significant difference between subgroups in the quality of fracture reduction (*P*>0.05).

**Table 1:** Operative information of patients in subgroups I and II of the study group.

**Operative information**	**Subgroup I (n=39)**	**Subgroup II (n=85)**	**Statistics**	***P*-value**
**Age (years, mean±SD)**	43.8±10.2	45.3±9.6	0.791	0.431
**Sex (*n* (%))**			0.173	0.677
Male	19 (48.7%)	38 (44.7%)		
Female	20 (51.3%)	47 (55.3%)		
**Sides**			0.017	0.897
Left	22 (56.4%)	49 (57.6%)		
Right	17 (43.6%)	36 (42.4%)		
**Injury mechanism**			2.350	0.503
Falls from standing	31 (79.5%)	67 (78.8%)		
Falls from a height	4 (10.3%)	11 (12.9%)		
Traffic accident	3 (7.7%)	7 (8.2%)		
Other	1 (2.6%)	0 (0)		
**Anatomical classification**			0.000	0.995
Subcapital	17 (43.6%)	37 (43.5%)		
Trans-neck	22 (56.4%)	48 (56.5%)		
**Garden’s classification**			2.791	0.095
Type III	29 (74.4%)	50 (58.8%)		
Type IV	10 (25.6%)	35 (41.2%)		
**Duration from the initial injury to operation (days, median (interquartile))**	2 (1)	3 (1)	-1.549	0.121
**Operative time (minute, mean±SD)**	81±7.4	82± 6.8	0.736	0.463
**Fluoroscopy time (second, mean±SD)**	18±2.1	19±1.7	2.833	0.005
**Blood loss (mL, mean±SD)**	194±31.5	203±23.6	1.592	0.117
**Quality of fracture reduction**			0.142	0.706
Garden index grade 1	22	51		
Garden index grade 2	17	34		

In subgroup I, two patients were lost to follow up and were excluded from the analysis of the functional outcomes. Thirty-seven patients were available for the final evaluation, and the average follow-up time was 26.1 months (range, 24-32 months) (Table 2). All fractures healed within an average of 3.4 months (range, 3 to 4 months). Two patients experienced AVN of the femoral head, and one presented a hematoma on the donor site of the iliac bone graft. The mean Harris score was 89.5 (range, 61-100). There were no significant differences in age, sex, follow-up period, or complication rates between patients younger than 50 years and those older than 50 years. However, patients younger than 50 years had higher Harris scores than those older than 50 years (*P*<0.05).

**Table 2:** Clinical outcomes of the patients in subgroups I and II of study group at the latest follow up.

	**Subgroup I (n=37)**			**Subgroup II (n=75)**		
**Age (years, patient no.)**	**≤ 50 yrs**	**> 50 yrs**	**total**	**≤ 50 yrs**	**>50 yrs**	**total**
	17	20	37	38	37	75
**Sex (n)**						
**Male**	11	7	18	24	9	33
**Female**	6	13	19	14	28	42
**Follow-up period (months, mean±SD)**	26.2±2.5	25.9±2.1	26.1±2.3	28.5±3.7	27.7± 3.6	28.1±3.6
**Harris score (mean±SD)**	94.7 ±6.9	85.1 ±11.1	89.5±10.7	90.7±8.3	85.5±16.0	88.1±11.6
**Complications**		3	3	5	4	9
**Avascular necrosis**		2	2	5	3	8
**Deep vein thrombosis**					1	1
**Hematoma on donor site**		1	1			

In subgroup II, seven patients were lost to follow up, two died from heart disease, and one died from liver cancer. Seventy-five patients were available for the final evaluation and were followed up for an average of 28.1 months (range, 24-34 months) (Table 2). All fractures healed within an average of 3.5 months (range, 3 to 5 months). Eight patients presented AVN of the femoral heads, and one experienced deep vein thrombosis. The mean Harris score was 88.1 (range, 41-100). There were no significant differences in age, sex, follow-up period, the Harris score, or the complication rate between patients younger than 50 years and those older than 50 years. In both subgroups (Table 2), the average duration from injury to surgery was 74 hours in ten patients with AVN of the femoral head and 46 hours in the other patients, with no significant difference (*P*=0.155). There was no significant difference in the Harris score between subgroups.

### The Control Group

The control group and study group were comparable in age, sex, fracture side, injury mechanism, fracture site, fracture classification and duration from the initial injury to operation (Table 3, all *P*>0.05). All fractures were treated by CRIF. The operative time and blood loss in control group were significantly less than those in study group (both *P*<0.05). There were no significant difference between groups in fluoroscopic time and the quality of fracture reduction (both *P*>0.05).

**Table 3:** Operative information of patients in study and control groups.

**Operative information**	**Study group (n=124)**	**Control group (n=96)**	**Statistics**	***P*-value**
**Age (years, mean±SD)**	44.8± 8.6	46.3±9.2	1.244	0.215
**Sex (*n* (%))**			0.083	0.774
Male	57 (46.0%)	46 (47.9%)		
Female	67 (54.0%)	50 (52.1%)		
**Sides**			0.100	0.752
Left	71 (57.3%)	57 (59.4%)		
Right	53 (42.7%)	39 (40.6%)		
**Injury mechanism**			0.895	0.827
Falls from standing	98 (79.0%)	75 (78.1%)		
Falls from a height	15 (12.1%)	12 (12.5%)		
Traffic accident	10 (8.1%)	9 (9.4%)		
Other	1 (0.8%)	0 (0)		
**Anatomical classification**			1.129	0.288
Subcapital	54 (43.5%)	35 (36.5%)		
Trans-neck	70 (56.5%)	61 (63.5%)		
**Garden’s classification**			0.208	0.648
Type III	79 (63.7%)	64 (66.7%)		
Type IV	45 (36.3%)	32 (33.3%)		
**Duration from the initial injury to operation (days, mean±SD)**	2.0±0.6	1.9±0.6	1.633	0.104
**Operative time (minute, mean±SD)**	82± 6.7	59±5.6	26.981	0.000
**Fluoroscopy time (second, mean±SD)**	19±1.8	14±2.4	16.882	0.000
**Blood loss (mL, mean±SD)**	200±31.7	92± 14.6	33.422	0.000
**Quality of fracture reduction**			0.297	0.586
Garden index grade 1	73 (58.9%)	53 (55.2%)		
Garden index grade 2	51 (41.1%)	43 (44.8%)		

Sixty-eight patients were followed up for an average of 31.2 months (range, 24-42 months) (Table 4). Patient age and sex were comparable between groups (both *P*>0.05). However, the follow-up period in study group was less than that in control group (*P*<0.05). The mean Harris score in control group was 83.8 (41–100), significantly lower than that in study group (*P*<0.05). The rates of AVN of the femoral head and fracture nonunion in control group were 26.5% (18/68) and 16.2% (11/68), respectively, significantly higher than those in study group (both *P*<0.05).

**Table 4:** Clinical outcomes of the patients in both groups at the latest follow-up.

	**Study group (n=112)**	**Control group (n=68)**	**Statistics**	***P*-value**
**Age (years, mean±SD)**	45.1±8.9	46.7± 10.1	1.111	0.268
**Sex (n)**			0.034	0.853
**Male**	51	30		
**Female**	61	38		
**Follow-up period (months, mean±SD)**	27.4±3.3	31.2± 5.1	5.781	0.000
**Harris score (mean±SD)**	88.6±12.7	83.8 ±13.3	2.415	0.017
**Avascular necrosis**	10 (8.9%)	18 (26.5%)	9.912	0.002
**nonunion**	0	11 (16.2%)	19.297	0.000

## Discussion

This study investigated the outcomes of acute displaced femoral neck fractures treated by CRIF augmented with free iliac bone block autografts with comparison to a routine protocol of CRIF without bone grafting. In study group, all fractures healed within five months. Patients in study group achieved better functional recoveries with less complications when compared to control group.

Displaced femoral neck fractures have a significant rate of poor outcomes due to a high incidence of complications, among which nonunion and AVN of the femoral head are the two most commonly encountered and intractable [13,14,24–30]. Displaced femoral neck fractures have been associated with a high incidence of nonunion. Despite advances in treatment, the reported incidence of nonunion after internal fixation ranges from 15% to 33% [31,32]. Park et al [33] analyzed 1133 femoral neck fractures retrospectively and reported a non-union incidence of 30.1% for displaced fractures v.s. a non-union rate of 8.5% for non-displaced fractures. Although nonunion occurs in younger patients to a greater degree, no correlation has been found among age, gender, or rate of nonunion [34]. The incidence of femoral head necrosis in displaced femoral neck fractures is also reportedly higher than that in non-displaced fractures [35]. Wei et al [35] retrospectively analyzed 222 femoral neck fractures and reported an AVN rate of 38.8% in displaced fractures *v.s.* an AVN rate of 3.2% in non-displaced fractures. In addition, Bonfiglio et al reported that osteonecrosis of the femoral head was highly associated with ununited fractures [36]. Therefore, we selected patients with displaced femoral neck fractures as subjects in this study to observe the effect of CRIF with free iliac bone block grafting. Non-displaced fractures were excluded from this study to reduce the risk of unnecessary grafting procedures to the utmost degree.

Various treatment algorithms, including muscle–pedicle bone grafting and vascularized or free bone grafting, have been attempted to reduce the complication rate of femoral neck fractures after internal fixation. Bone grafting with internal fixation has emerged as a reliable method with good long-term functional outcomes [37]. Meyers et al [19] treated displaced subcapital and transcervical fractures of the femoral neck by muscle-pedicle-bone grafting and internal fixation in an open manner. They reported a nonunion rate of 11% in 130 patients followed for six months or more. However, the technique of vascularized pedicle grafting is highly technical and requires microsurgical facilities and experience. Free iliac bone grafting is easy to perform and can achieve a satisfactory result when treating nonunion of a femoral neck fracture or a neglected fracture. To reduce the iatrogenic damage of an open operation and make the bone grafting easier to perform, we treated acute displaced femoral neck fractures using free iliac bone block grafting in a minimally invasive fashion.

Displaced femoral neck fractures often accompany comminution of the posteromedial cortex of the femoral neck, which is an important risk factor of nonunion due to the loss of the buttressing effect against lateral rotation and insecure fixation [27,38–43]. There is a good correlation between fracture stability and uneventful healing of femoral neck fractures [44]. It is vital to provide rigid structural support after anatomical reduction of a femoral neck fracture is achieved. In the current study, the iliac bone block graft was comprised of tricortical bone and the intermediate cancellous bone. Cancellous bone plays a significant role in osteoinduction and osteogenesis and, therefore, in improving the healing of fractures and nonunion. The cortex of the iliac graft can provide structural support for a fractured femoral neck, which can improve biomechanical stability and prevent late-stage collapse. In addition, femoral neck fractures were fixed with three parallel cannulated screws. Inserting screws parallel to each other can provide compression across the fracture site and improve the overall stability of fixation [45–50], which has a significantly lower nonunion rate than vertical- and separated-type screw configurations [42]. Harper et al [51] reported that the intracapsular pressure will increase after femoral neck fracture, blocking venous return and increasing intraosseous pressure, which will subsequently lead to femoral head necrosis. In study group, the tunnel created along the femoral neck decreased the intraosseous pressure and improved the venous return, subsequently decreasing the risk of femoral head necrosis. In total, 112 patients were available for the final evaluation, and all fractures had united in study group. Although AVN of the femoral head was detected in ten patients (8.9%), no collapse occurred in these cases during a greater than two-year follow up. In control group, the rates of fracture nonunion and AVN of the femoral head were 16.2% and 26.5%, respectively. The rates of nonunion and AVN in study group were much lower compared to those in control group and previous reports.

A major limitation is that this technique adds an additional operative injury to all patients with displaced femoral neck fractures, although iliac bone block grafting is a commonly applied and simple procedure with less invasion and few complications. Not all patients will sustain nonunion or femoral head osteonecrosis. We have previously investigated high-risk factors for ununited femoral neck fractures [52]. In a subsequent study, this technique will only be applied to patients who are at great risk of sustaining nonunion or AVN of the femoral head. Another limitation is that patients in control group were followed up longer than those in study group, which may have an influence on comparion of the rate of AVN of the femoral head between groups. The third limitation is that patients older than 60 years (<65 years old) were enrolled in this study. For the elderly, it remains a matter of debate whether displaced femoral neck fractures should be treated by internal fixation or arthroplasty. Some surgeons suggest that older patients (>60 years) with displaced femoral neck fractures should receive hemi-arthroplasty or total hip replacement [53–55]. However, other surgeons recommend internal ﬁxation for patients younger than 65 years with displaced fractures of the neck of the femur [56–61]. In our cilinical pratice, internal fixation is commonly the treatment of choice in patients younger than 65 years old, as this treatment allows for preservation of native bone [60].

## Conclusion

We treated acute displaced femoral neck fractures by CRIF with free iliac bone block grafting. This technique is easy to perform with less invasion, which can guarantee uneventful fracture healing and significantly reduce the incidence of osteonecrosis of the femoral head. This technique is an effective alternative for orthopedists to treat displaced femoral neck fractures.
